# Effect of *Ananas comosus* nanoemulgel on traumatic ulcers in the inflammatory phase

**DOI:** 10.1016/j.jtumed.2025.02.016

**Published:** 2025-03-13

**Authors:** Juni Handajani, Widjijono, Heni Susilowati, Yesica Dea Cahyani, Shafira Zakia Rahma

**Affiliations:** a Department of Oral Biology, Faculty of Dentistry, Universitas Gadjah Mada, Indonesia; b Department of Dental Material, Faculty of Dentistry, Universitas Gadjah Mada, Indonesia; c Student of Magister Dental Science Study Program, Faculty of Dentistry, Universitas Gadjah Mada, Indonesia

**Keywords:** بروميلين, أناناس, المرحلة الالتهابية, العدلات, كوكس-۲., Bromelain, COX-2, Inflammatory phase, Neutrophils, Pineapple (*Ananas comosus* (L.))

## Abstract

The initial stage of the wound healing process is the inflammatory phase involving cellular and molecular mechanisms. Traumatic ulcers can develop due to injuries to the oral mucosa. Natural ingredients could potentially be used as medicines to treat wounds in the oral cavity, including pineapple extract containing bromelain enzymes.

**Objectives:**

This study evaluated the effects of applying pineapple extract (*Ananas comosus* (L.) Merr) nanoemulgel on the inflammatory phase in the traumatic ulcer healing process.

**Methods:**

Twenty-four male Wistar rats aged 2–3 months were injured with a punch biopsy (Ø 3 mm) in the buccal mucosa. Subjects were randomly assigned to a positive control group (Aloclair Plus Gel®) and treatment group (pineapple extract nanoemulgel), where each group contained 12 rats. Gel was applied to the surface of the wound in the buccal mucosa each day for 4 days. Four rats were sacrificed on days 0, 1, 2, and 3, and specimens were prepared for histological assessment. Hematoxylin and eosin staining was used to assess the number of neutrophils and immunohistochemical staining to determine COX-2 expression. Data were analyzed by analysis of variance (ANOVA) and using the least significant difference test (LSD).

**Results:**

The results showed that compared with the control, the wound diameter was significantly smaller in the treatment group and the number of neutrophils and COX-2 expression level were significantly higher (*p* < 0.05). The number of neutrophils peaked on day 1 and the COX-2 expression level on day 3. These findings indicate that pineapple stem extract could accelerate wound healing.

**Conclusion:**

Following induction of a traumatic ulcer with a small diameter, a greater number of neutrophils and higher COX-2 expression level were found in the group treated using pineapple extract nanoemulgel compared with the control.

## الملخص

**أهداف البحث:**

المرحلة الالتهابية هي المرحلة الأولية في عملية التئام الجروح والتي تتضمن آليات خلوية وجزيئية. تعرف القرحات الرضحية بأنها إصابات في الغشاء المخاطي للفم. يستمر تطوير المكونات الطبيعية لعلاج الجروح، بما في ذلك استخدام مستخلص الأناناس مع محتوى إنزيم البروميلين، والذي يعتقد أنه يتمتع بإمكانات كدواء للجروح في تجويف الفم. تهدف هذه الدراسة إلى تحديد وتقييم تأثيرات نانو إمولجيل الأناناس على المرحلة الالتهابية في عملية التئام القرحات الرضحية.

**طريقة البحث:**

تم إصابة أربعة وعشرين من ذكور جرذان ويستار وعمرها من شهرين إلى ثلاثة أشهر بخزعة على الغشاء المخاطي للفم. تم تقسيم المشاركين عشوائيا إلى مجموعة تحكم إيجابية (ألوكلير بلس جل) ومجموعة علاج (نانو إمولجيل الأناناس)، كل منها تضم 12 فأرا. تم تطبيق الجل عن طريق تطبيق الجل بعرض سطح الجرح المخاطي الخدي الذي تم إجراؤه مرة واحدة يوميا لمدة ٤ أيام. في الأيام صفر و۱ و۲ و٣، تم التضحية بالجرذان بمعدل ٤ يوميا، تلا ذلك تحضيرات علم الأنسجة. تم استخدام صبغة الهيماتوكسيلين إيوزين لتحديد عدد الخلايا المتعادلة، وتم استخدام الصبغة المناعية الكيميائية لتحديد تعبير كوكس-۲. تم تحليل البيانات باستخدام تحليل التباين.

**النتائج:**

أظهرت النتائج أن قطر الجرح كان أصغر بشكل ملحوظ في مجموعة العلاج مقارنة بالمجموعة الضابطة، في حين كان عدد الخلايا المتعادلة وتعبير كوكس-۲ أعلى بشكل ملحوظ. بلغ عدد الخلايا المتعادلة ذروته في اليوم الأول، بينما كان التعبير عن كوكس-۲ في اليوم الثالث. وخلصت الدراسة إلى أن مستخلص جذع الأناناس يمكن أن يسرع من التئام الجروح.

**الاستنتاجات:**

تم الإشارة إلى ذلك سريريا من خلال قطر قرحة رضية أصغر، وعدد أكبر من الخلايا المتعادلة، والتعبير عن كوكس-۲ في مجموعة العلاج مقارنة بالمجموعة الضابطة**.**

## Introduction

In the initial stage of the wound healing process, an inflammatory reaction occurs involving various cellular and molecular mechanisms. The inflammatory phase consists of early inflammation (hemostasis phase), which occurs immediately after the wound is made (day 0), and late inflammation, which lasts until day 5 after the wound is made. However, other studies state that the inflammatory phase usually lasts from day 1–3 after the wound is made.^1^^,^^2^

Oral mucosal wounds occur often in individuals due to the loss or damage of part of the oral mucosal tissue, which can be caused by physical trauma, such as incorrect tooth brushing, careless use of toothpicks, sharp pieces of food, and hot food or drinks.^3^ Wounds on the oral mucosa differ from those on the body's surface because of their different anatomical structures. In the oral cavity, mucosal tissue is always kept moist with saliva, whereas the dry surface of the body is covered by skin, with sweat glands, oil glands, and fine hairs.^4^ Nagy et al.^5^ stated that saliva is important for wound healing because it contains epidermal growth factor, which plays a role in the wound healing process. Furthermore, Nagy et al. concluded that deficiency of this growth factor in the saliva is closely related to decreased efficiency of the wound-healing process in the oral cavity.^5^

Traumatic ulcers can be caused by wounding of the oral mucosa due to mechanical, thermal, chemical, and electrical trauma. According to Prasetyaningrum and Ardana,^6^ approximately 83.6 % of people have experienced traumatic ulcers on the oral mucosa. Traumatic ulcers are characterized by loss of epithelium in the middle of a yellowish fibrin exudate with erythematous edges, and they can have a round shape and differ in size depending on the duration, intensity, and cause.^7^ Wounds on the mucosa induce COX-2 mRNA and protein expression in the basal layer of the epidermis, peripheral cells near hair root follicles, fibroblasts, and capillaries near the epidermis at the wound's edge.^8^ COX-2 expression is dependent on stimulation in peripheral tissue that is inflamed, or other pathological conditions. The COX-3 enzyme acts as a catalyst in the formation of inflammatory mediators, such as prostaglandins and thromboxanes.^9^

The oral mucosa healing process involves complex mechanisms and interconnected stages. The process begins with bleeding caused by tissue damage. Blood fills the injured tissue, followed by platelet degranulation and the activation of Hageman factor. The subsequent process consists of inflammatory, proliferation, and remodeling phases, before epithelial migration near the wound, fibroblast proliferation, and capillary regeneration to produce new granulation tissue.^1^^,^^2^^,^^10^

The inflammatory process is required to eliminate or limit the causative agent of injury and restore damaged oral mucosa. The inflammatory phase is an important initial process that triggers wound healing,^2^ where it is characterized by brief vasoconstriction in blood vessels, followed by local vasodilation of blood vessels, resulting in increased blood flow and capillary permeability, accompanied by the release of plasma proteins and neutrophils into the tissue. Neutrophils released in the inflammatory phase are essential for eliminating bacteria or the causative agents of injury. However, excessive release of neutrophils should not be allowed to continue because they will inhibit the wound healing process.^1^^,^^2^

The development of medicinal materials from natural ingredients continues to be important, including pineapple (*Ananas comosus* (L.)). In general, only the pineapple fruit's flesh is consumed, whereas other parts, such as the crown, skin, and tuber, are not utilized optimally. The pineapple stem contains various active substances such as bromelain enzymes, tannins, flavonoids, saponins, alkaloids, tacorin, and glycosides.^11^^,^^12^ Bromelain enzymes can be found in all parts of the pineapple plant, but the highest content is found in the stem.^13^ Bromelain enzymes are considered to have potential applications in wound healing due to their anti-inflammatory, anti-invasive, and anti-metastatic effects. Studies of the effects of bromelain enzymes on wound healing indicate that these enzymes can accelerate the maturation of granulation tissue in cuts and re-epithelialization during the healing of burns. In vivo studies demonstrated that a bromelain concentration of 1 % also obtained good results in accelerating the healing of burns, as indicated by a decrease in the wound diameter, and complete closure of the wound on day 15 of observations.^4^^,^^14^, ^15^ In the present study, we evaluated the effects of pineapple stem extract (*Ananas comosus* (L.) Merr) nanoemulgel on the inflammatory phase during the traumatic ulcer wound healing process. The results obtained in this study may facilitate the development of natural organic materials from pineapple stem to aid the healing of traumatic ulcer wounds on oral mucosa.

## Materials and Methods

Ethical clearance was obtained from the Dental Research Ethics Commission of the Faculty of Dentistry and Prof. Soedomo Hospital of Gadjah Mada University through letter No. 69/UN1/Kep/FKG-RSGM/EC/2024 dated April 30, 2024. Pasir Kelud pineapple was provided by the Langgeng Mulyo Agricultural Cooperative, Kediri, East Java, Indonesia. Plant determination was carried out at the Plant Systematics Laboratory of the Faculty of Biology, Universitas Gadjah Mada, through letter No.00614/S.Tb./V/2024 dated May 6, 2024, and the plant material was confirmed as: family Bromeliaceae, genus *Ananas*, species *Ananas comosus* (L) Merr.

### Preparation of pineapple stem extract

Pineapple stem extraction was conducted at the Pharmacognosy Laboratory of the Surabaya Academy of Pharmacy. The pineapple was yellowish green at 18 months and the stem weighing 916.48 g was used for extraction. The stem was cleaned, washed, cut into small pieces, and heated at a temperature of 45 °C for 15 min. After heating, the pineapple stem was homogenized in a blender by adding 1000 mL of phosphate buffer solution (pH 7.0), before filtering and storing the filtrate (designated as pineapple stem extract) at a temperature of 4 °C.

### Preparation of pineapple stem extract nanoemulgel

Pineapple stem extract nanoemulgel was prepared at the Pharmaceutical Laboratory of the Surabaya Academy of Pharmacy by using virgin coconut oil (Parchem, USA), Tween 80 (Croda, UK), Span (Croda, UK), carbomer (Oxford, India), triethylamine (Chemistry Connection, USA), nipagin (Clariant, Mutierz, Switzerland), and pineapple stem extract at a concentration of 1 %.

The bromelain content of the pineapple stem extract was measured at the Instrument Laboratory of the Bhakti Wiyata Kediri Health Science Institute. The bromelain content was determined using the biuret method.^16^

The physical stability of the pineapple stem extract nanoemulgel material was tested based on its organoleptic properties (color, odor, and appearance), homogeneity, pH, spreadability, and particle size. Particle size testing was carried out using a particle size analyzer (Nanotrac Wave II, Japan) at the Pharmaceutical Laboratory of the Surabaya Academy of Pharmacy.

### Treatment of experimental animals

Twenty-four Wistar rats aged 2–3 months and weighing 250–300 g were used as research subjects. They were adapted in individual cages for 3 days. The Wistar rats were then randomly assigned to the treatment group and control group, where each contained 12 rats. Tests with experimental animals were performed at the Integrated Research and Testing Laboratory, Universitas Gadjah Mada.

Traumatic ulcers were induced by first anesthetizing the rats with a dose of 0.1 mL/100 g of body weight ketamine (Ketamil, CV. Karebet Karya Persada, Indonesia, catalog No. 2960000999-2HH-100335784) intramuscularly,^17^ before making wounds using a punch biopsy (Ø 3 mm, Medax model Epitheasy, Italy) by pressing and rotating on the buccal mucosa. Gel was applied once each day in the morning for 4 days using a regular size 3.0 mm microbrush® (Microbrush International, Wisconsin, USA) for rats in the treatment and control groups. Rats in the control group were treated using Aloclair Plus® gel (Kalbe Farma, Indonesia).

Decapitation was performed on days 0, 1, 2, and 3 after rats were anesthetized intramuscularly using ketamine HCl at a dose of 0.1 mL/100 g of body weight.^17^ Ulcer tissue was sampled from the buccal mucosa and a biopsy excision was made with a thickness of ± 2–3 mm. The tissue was fixed by immersing in 10 % buffered natural formalin solution for 24 h.

Aseptic technique was applied to avoid wound infection by sterilizing all instruments, using sterile gloves, masks, and gowns, and ensuring that the surgical area was clean and disinfected. Hygienic disposal of rats was performed after completing the experiment according to the standard operating procedure of the Integrated Research and Testing Laboratory, Universitas Gadjah Mada.

### Preparation of histological specimens

Histological specimens were prepared at the Integrated Research Laboratory of the Faculty of Dentistry, Gadjah Mada University. The ulcer tissue specimens were washed with water to remove the remaining fixative solution, before dehydration using a graded series of alcohol concentrations, (i.e., 70 %, 80 %, 96 %, and 100 %), for 20 h in an automatic tissue processor (Tissue-Tek II Sakura Timing Disc, USA). The specimens were then cleared using xylol, before paraffin infiltration and embedding in a tissue embedding cassette (refurbished Sakura® TEC^TM^ 4 tissue embedding system, USA). Tissue samples were cut to a thickness of 3 μm using a microtome (Accu-Cut® SRMTM Rotary Microtome, Sakura FineTek Co., Ltd, Japan).

### Tissue staining

Tissue samples were stained using hematoxylin and eosin (Harris Hematoxylin, Leica Biosystems, USA), and immunohistochemical stain (Elabscience, USA). Deparaffinization and rehydration procedures were performed using graded xylol and alcohol solutions ranging from 100 % down to 95 %, 80 %, and 70 %.

Histological staining was conducted by immersing the specimens in hematoxylin solution for 5 min and rinsing with running water for 3 min, before staining with eosin solution for 30 s and rinsing with running water. The tissue samples were then dehydrated using graded alcohol solutions with concentrations of 70 %, 80 %, 96 %, and 100 %, before clearing using xylol and mounting.

Immunohistochemical staining was performed using an IHC kit (Elabscience, USA). Peroxidase blocking was conducted by dripping 3 % H_2_O_2_ onto the specimen and incubating at room temperature for 10 min to eliminate endogenous peroxidase activity, before washing with phosphate-buffered saline (PBS). Drops of normal goat blocking buffer were applied to the specimens and incubated for 30 min at 37 °C, followed by incubation overnight at 4 °C with 50 μL of primary antibody anti-COX-2 (rabbit polyclonal antibody COX-2, NB100-689, Novusbio, USA) at 1:100. The specimen was washed with PBS and incubated with polyperoxidase-anti-mouse/rabbit IgG (E-IR-R217B) for 20 min at room temperature or 37 °C, before washing with PBS. Staining was conducted using the chromogen 3,3′-diaminobenzidine at a ratio of 1:20 in the substrate until a yellow-brown color appeared as a positive sign. The process was completed by dehydrating using a series of graded alcohol solutions, clearing using xylene, and mounting.

### Observation of neutrophil number and immunohistochemistry

The numbers of neutrophil cells in the connective tissue at the edge of the wound area were counted using a light microscope under magnification at 400× and wound area was observed under magnification at 40x. Three fields of view were observed for each prepared specimen. The result was then calculated as the average number of neutrophils in each field of view.

COX-2 protein expression was observed in the basal layer of the epidermis and connective tissue at the edge of the wound area. The expression of COX-2 was characterized based on observing brown color in the cytoplasm of neutrophil cells, macrophages, lymphocytes, fibroblasts, and extracellular matrix. COX-2 expression was measured in the epidermis area at the edge of the wound based on three fields of view under magnification at 400×. Evaluations of photographic images of specimens were conducted using free access Image J software, version 154 (National Institutes of Health, USA). The results were then analyzed using parametric statistical tests (ANOVA and LSD).

## Results

The stability of the gel material was assessed by testing its organoleptic properties, homogeneity, pH, spreadability, adhesion, and particle size. Organoleptic evaluations based on color, odor, and appearance parameters showed that the gel was white with a distinctive aromatic odor and thick consistency (gel). Homogeneity analysis indicated that the material was homogeneous with no coarse grains. The degree of acidity (pH) was in the range of 6.29–6.33. A good degree of acidity (pH) is close to neutral (6–7) for the mouth area. The average evaluation of spreadability was 5.03 cm. The criterion for topical use of a good semi-solid preparation is spreadability of around 5–7 cm in diameter. The adhesion test results indicated a range of 4.12–4.23 s, thereby satisfying the requirement for good adhesion of not less than 4 s. Testing using a particle size analyzer demonstrated that the average particle size was 260.65 nm, which met the requirement for nanoparticle size, that is, a range of 10–1000 nm.

**Figure 1:**
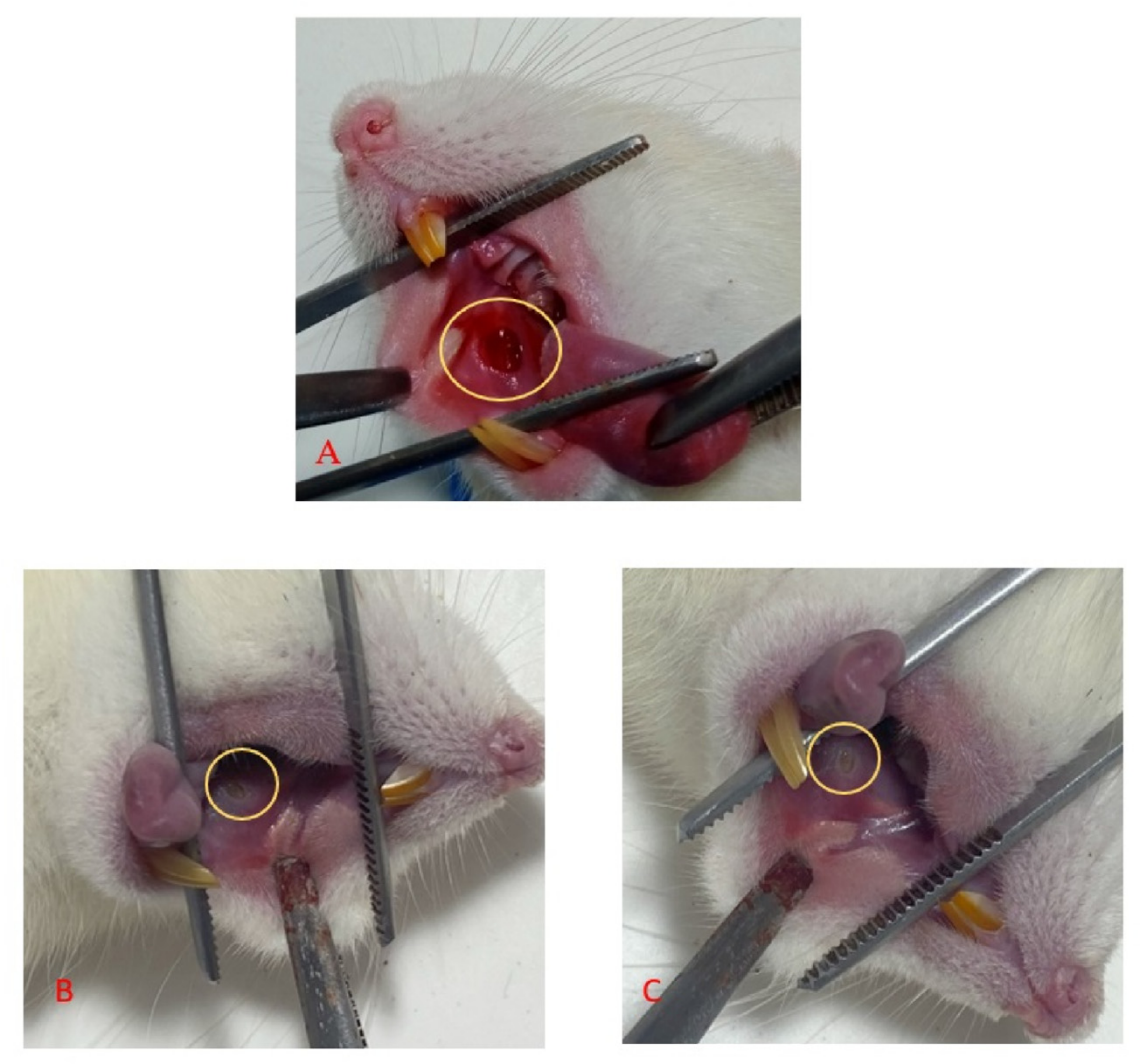
Clinical images of traumatic ulcers on the buccal mucosa of rats. An ulcer with a diameter of Ø 3 mm was observed on day 0 (A). On day 3, the diameters of the ulcers were similarity decreased in the treatment (pineapple stem extract) (B) and the control (C) groups.

Figure 1 shows clinical observations of the buccal mucosa after applying pineapple stem extract. The traumatic ulcers were almost closed in the treatment and control groups on day 3.

**Figure 2:**
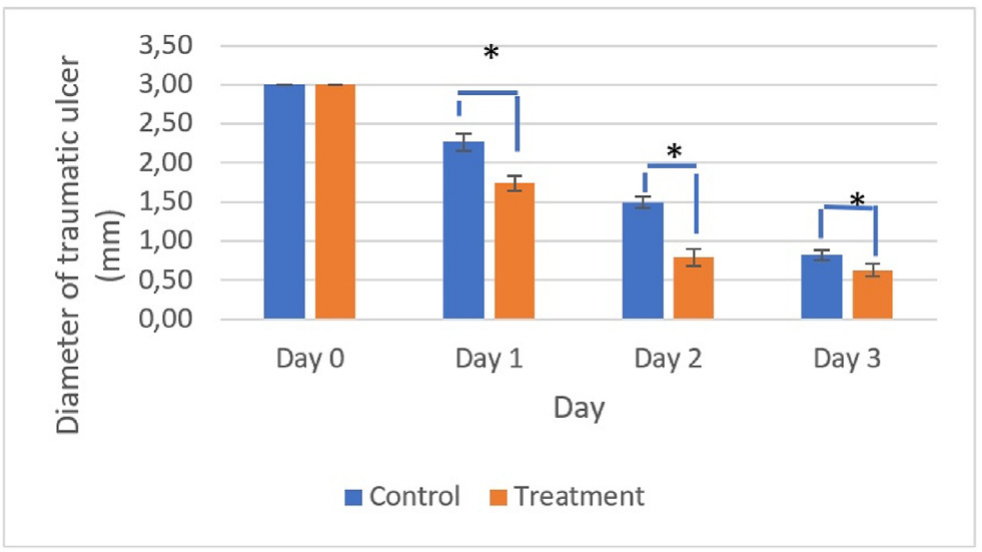
Decreases in ulcer diameters over time. The diameters of the ulcers were significantly smaller in the treatment group than the control group (∗, *p* < 0.05).

The diameters of the traumatic ulcers on days 0–3 are shown in Figure 2.

**Figure 3:**
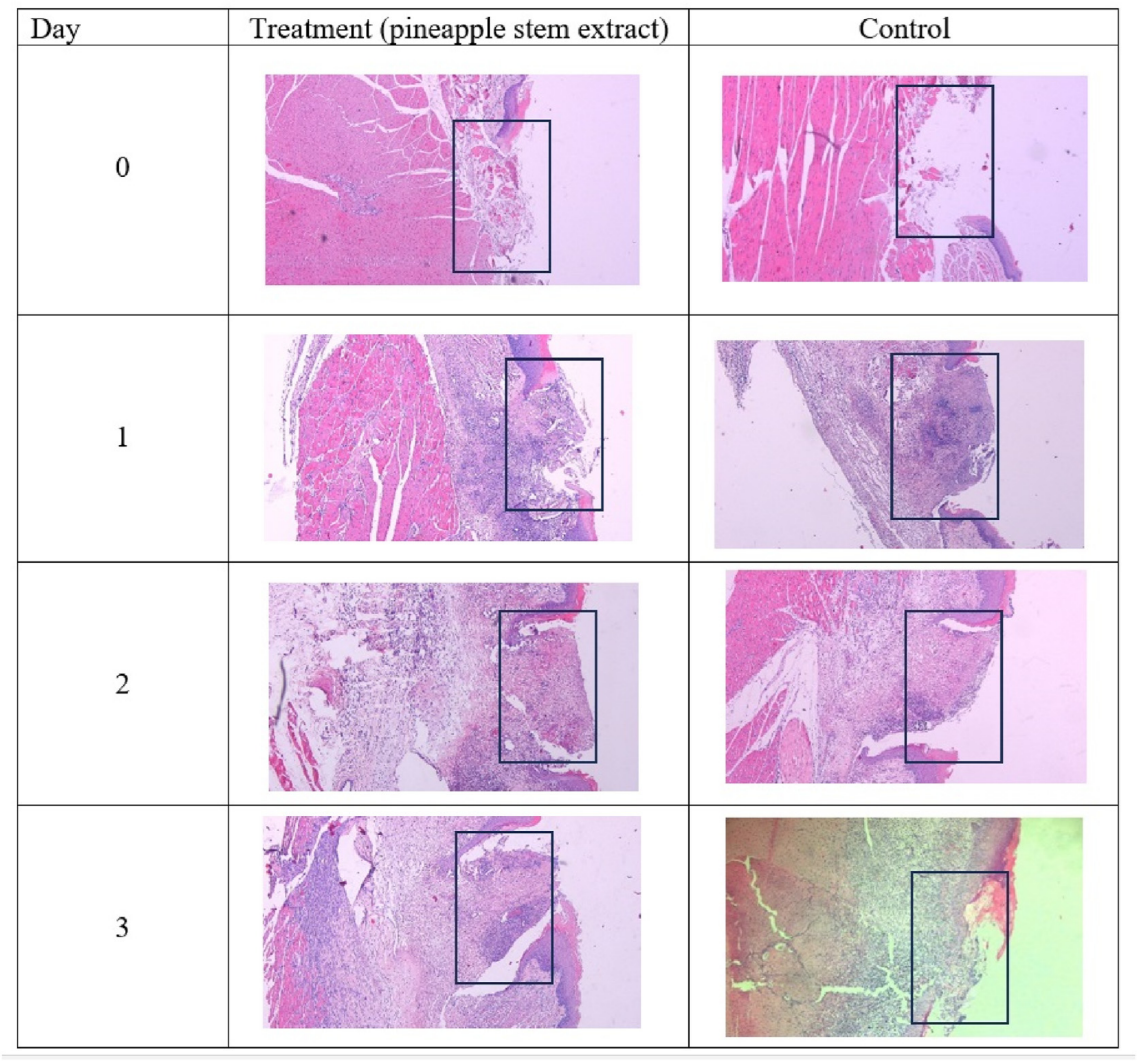
Evaluation of the wound healing process in the inflammatory phase (day 0–3) after applying pineapple extract (A) and control (B) treatments. Inflammatory cell infiltration was observed on day 1 and began to decrease on day 3, while the wound diameter decreased each day. Hematoxylin and eosin staining at 40× magnification.

Figure 3 shows histological images of the traumatic ulcer wound healing processes in the treatment and control groups during the inflammatory phase.

Histological images were obtained to observe the wound healing processes after applying pineapple stem extract nanoemulgel and Aloclair Plus® gel control. Inflammatory cell infiltration was not observed on day 0, but dense inflammatory cells were detected on day 1. The wound diameters decreased on day 3.

**Figure 4:**
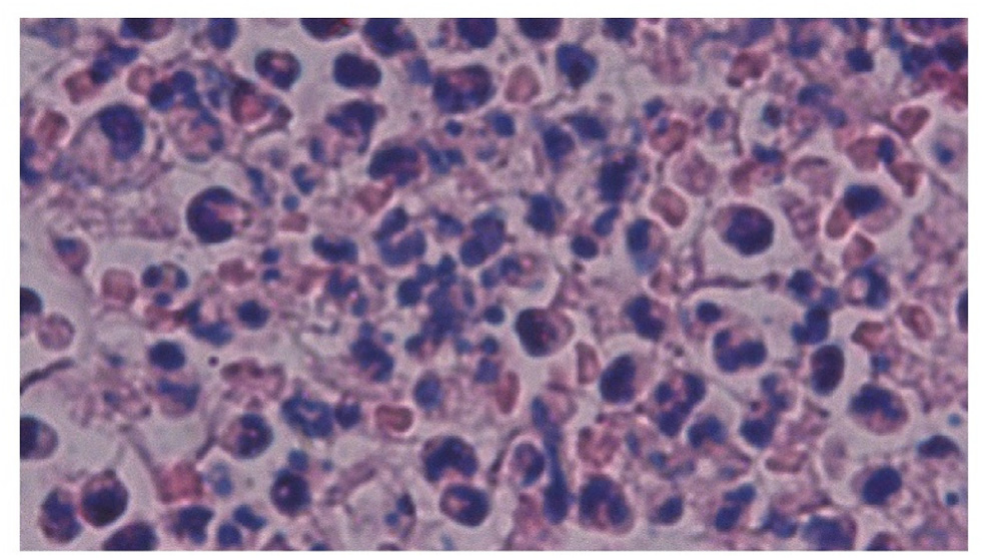
Histological observation of traumatic ulcer on day 1 showing inflammatory cell infiltration dominated by neutrophil cells. Hematoxylin and eosin staining at 1000× magnification.

**Figure 5:**
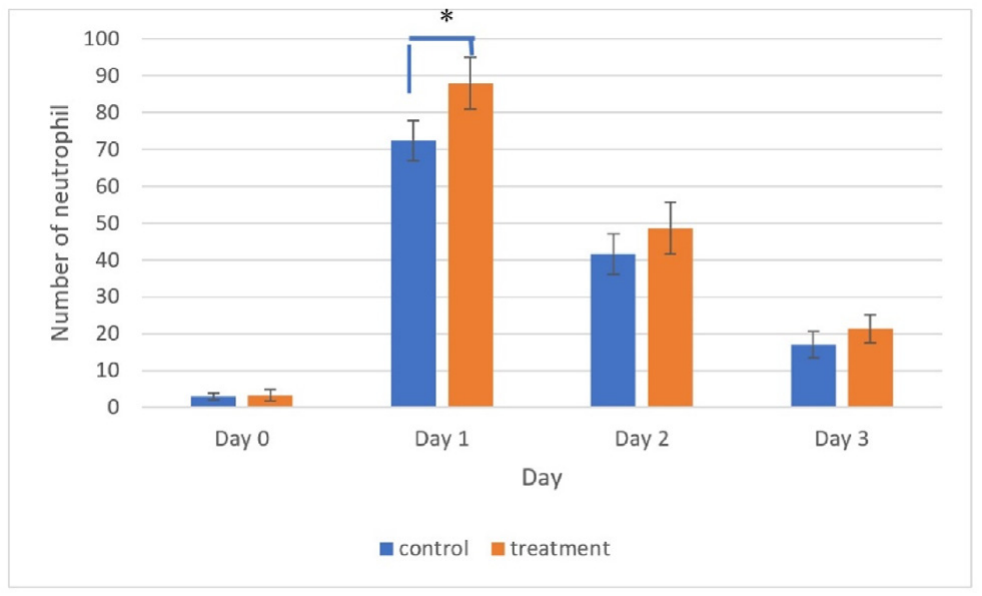
The number of neutrophils peaked on day 1 in both the treatment and control groups, before decreasing subsequently. The number of neutrophils was significantly higher in the treatment group than the control group (∗, *p* < 0.05).

Inflammatory cell infiltration was observed on day 1 and dominated by neutrophils (Figure 4). The number of neutrophils peaked on day 1 in both the treatment and control groups, before decreasing subsequently (Figure 5).

**Figure 6:**
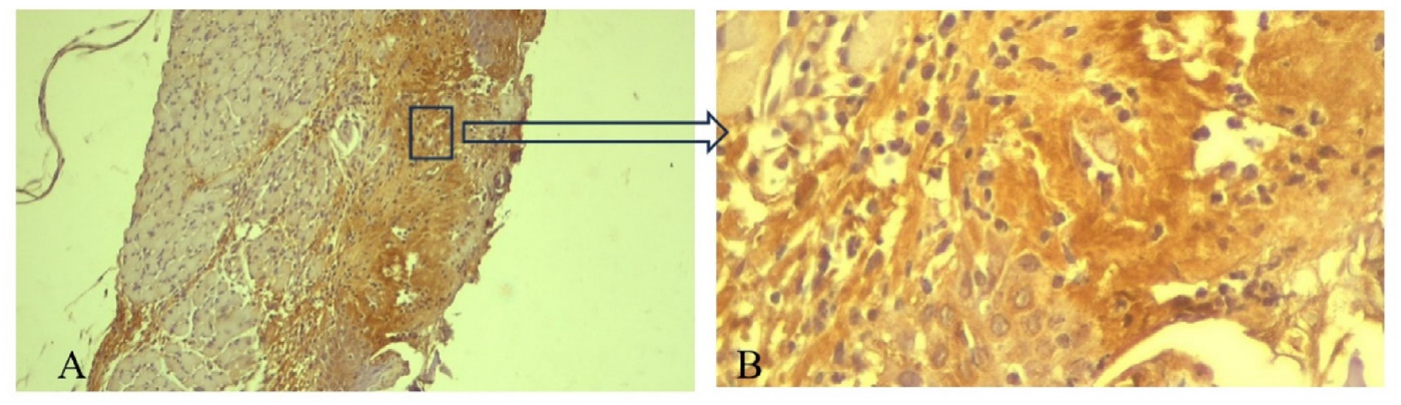
Positive expression of COX-2 was visualized by brown staining in the cytoplasm of inflammatory cells and extracellular matrix in the ulcer area. Immunohistochemical staining on day 3 in the treatment group with pineapple stem extract application. Magnification 100× (A) and 400× (B)

**Figure 7:**
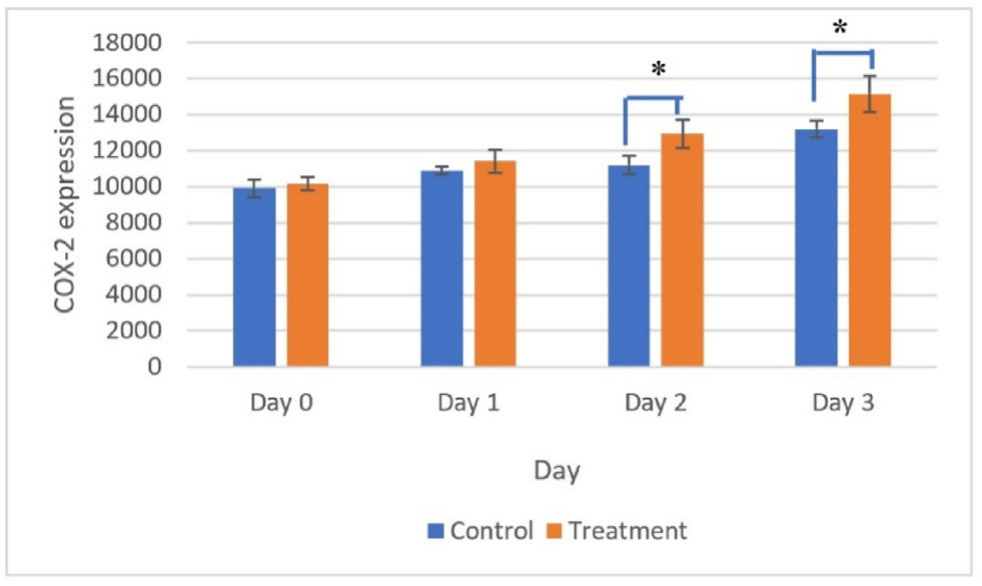
COX-2 expression peaked on day 3 in both treatment and control groups. COX-2 expression was significantly higher in the treatment group than the control group on days 2 and 3 (∗, *p* < 0.05).

Positive COX-2 expression was visualized based on brown staining in the cytoplasm of neutrophil cells, macrophages, lymphocytes, fibroblasts, and the extracellular matrix (Figure 6). COX-2 expression peaked on day 3 in both the treatment and control groups (Figure 7).

Tests of normality and homogeneity conducted using the Shapiro–Wilk and Levene tests indicated that the numbers of neutrophils and COX-2 expression levels were normally distributed and homogeneous (*p* > 0.05). The ANOVA results detected significant differences in the number of neutrophils and COX-2 expression levels between treatments (*p* < 0.05). The results obtained using the LSD test showed that the number of neutrophils on day 1 was significantly higher in the treatment group compared with the control group. By contrast, the COX-2 expression levels on days 2 and 3 were significantly higher in the treatment group than the control group. These results indicate that treatment with pineapple stem extract accelerated the healing of traumatic ulcers characterized by a small wound diameter compared with the control. Pineapple stem extract triggered neutrophil infiltration on day 1 and induced COX-2 expression on day 3.

## Discussion

In the present study, treatment with pineapple stem extract induced the inflammatory phase in the traumatic ulcer healing process. In particular, in the inflammatory phase of the oral mucosal ulcer healing process, the number of neutrophils increased sharply on day 1 after injury and then decreased until day 3. Starting from day 1, the number of neutrophils was higher in the treatment group than the control group. COX-2 expression increased until the peak was reached on day 3. COX-2 expression was higher in the treatment group than the control group.

According to Landén et al.,^18^ the inflammatory induction mechanism involves blood vessels breaking in the wound area, causing bleeding. The bodily response includes the activation of extrinsic and intrinsic coagulation factors to stop the bleeding. Furthermore, hemostasis occurs because blood from the injured area comes into contact with collagen and the extracellular matrix, thereby triggering the release of platelets (thrombocytes).^18^ Platelet cells undergo degranulation to release cytokines and activate extrinsic and intrinsic pathways that stimulate neutrophil cells to migrate to the provisional matrix and initiate the inflammatory phase.^18^ Cytokines secreted by platelet cells function to promote the secretion of inflammatory factors and release of various growth factors, such as vascular endothelial growth factor, platelet-derived growth factor, insulin-like growth factor-1, transforming growth factor-β, epidermal growth factor, interleukin-1, cytokines, and chemokines.^19^

In the present study, pineapple stem extract was used as the treatment because it contains various active substances, although the stem is generally considered waste material. The active substances in pineapple tubers include bromelain enzymes, tannins, flavonoids, saponins, alkaloids, tacorin, and glycosides,^11^ which may have roles in wound healing. The most abundant active substances in pineapple tubers are bromelain enzymes.^20^ A study by Ilyas et al.^21^ showed that the specific activity of pineapple stem is higher than that of pineapple flesh, and that pineapple tubers are resistant to high temperatures.

The results obtained in the present study showed that applying pineapple stem extract as the treatment could stimulate neutrophil infiltration and induce COX-2 expression. Figure 4, Figure 5 show that inflammatory cell infiltration on day 1 was dominated by neutrophils, and the number of neutrophils was significantly higher in the treatment group. These results may have been influenced by the active ingredients in pineapple stems, especially bromelain enzymes. Bromelain enzymes are proteolytic enzymes that can catalyze hydrolysis reactions to break proteins down into simpler peptides. These enzymes can reduce the pain and swelling caused by wounds or injuries. Bromelain enzymes are used in therapeutic applications, such as antiplatelet, antithrombotic, platelet aggregation, fibrinolytic, anticancer, anti-inflammatory, and surgical trauma treatments, and for debridement in wound healing.^15^^,^^22^^,^^23^ Pineapple stem also contains flavonoids, tannins, and saponins with antioxidant and anti-inflammatory properties. Flavonoids could inhibit the production of inflammatory mediators, while tannins and saponins may reduce inflammation by inhibiting the activity of the inflammatory process.^24^ The anti-inflammatory effect of pineapple stem extract was characterized by the dominance of neutrophil infiltration on day 1.

Figure 1, Figure 2, Figure 3 show that the healing of ulcer wounds was characterized by decreases in the ulcer diameters in both the treatment and control groups; however, the ulcer diameter was significantly smaller in the treatment group compared with the control (Figure 2), possibly due to the effects of bromelain enzymes on healing ulcers through proteases hydrolyzing fibrin clots in wounds. Bromelain enzymes are also known to repair damaged extracellular matrix components such as collagen, laminin, and elastin through proteolysis effects, thereby inducing the release of growth factors and angiogenesis.^25^

The expression of COX-2 peaked on day 3 in both the control and treatment groups. COX-2 expression was found to increase until day 3 even though the numbers of neutrophils decreased and the diameters of the oral mucosal wounds decreased. The anti-inflammatory mechanism associated with bromelain enzymes is still unclear. Bromelain enzymes might not be COX-2 inhibitors, but instead they may lead to anti-inflammatory effects through various mechanisms, including reducing the production of pro-inflammatory cytokines and modulating immune cell activities.^25^

In the control group, we applied one drop of Aloclair Plus® gel to the oral traumatic ulcer. The healing of ulcer wounds is attributed to the active ingredients in Aloclair Plus®, which have anti-inflammatory effects. The active ingredients in the control treatment included *Aloe vera*, sodium hyaluronate, glycyrrhetinic acid, and polyvinylpyrrolidone (PVP). These four ingredients may reduce inflammation, especially PVP, which can form a layer on the wound's surface to prevent further irritation of the ulcer.^26^

In the present study, 1 % nanoemulgel pineapple stem extract was used as the treatment. According to a previous study conducted by Thomas et al., a 1 % concentration of pineapple bromelain led to rapid wound recovery, as indicated by the decreased diameter of a burn, and the wound closed completely on day 15.^15^ Nanoemulgel was prepared from pineapple stem extract because the nanoparticle form could carry hydrophilic and hydrophobic therapeutic ingredients. Gel preparations can also dissolve more rapidly compared with cream or ointment preparations.^27^ Nanoemulgel is a suitable dosage form for delivering therapeutic ingredients through the skin.^28^

The results obtained in the present study demonstrate that pineapple stem extract can be applied as a therapeutic ingredient to accelerate the healing of traumatic ulcers on the oral mucosa. The number of neutrophils and COX-2 expression levels were high on day 3, and the wound diameter decreased. A limitation of this study is the need to identify an optimal pineapple stem extract formulation for use as an oral mucosal therapy. In addition, longer term observations are still needed to understand the overall oral mucosal wound healing process.

## Conclusion

Pineapple stem extract induced the inflammatory phase and accelerated wound healing, as indicated clinically by the decreased diameter of traumatic ulcers, higher neutrophil count, and greater COX-2 expression level in the treatment group compared with the control group.

## Ethical approval

This study was approved by the FKG-RSGM UGM, Indonesia Ethics Committee under reference number 69/UN1/Kep/FKG-RSGM/EC/2024.

## Authors contributions

JH and HS conceived and designed the study, conducted research, provided research materials, and collected and organized data. JH and W analyzed and interpreted data. JH, HS, YDC, and SZR wrote the initial and final drafts of the article. All authors critically reviewed and approved the final draft, and are responsible for the content of the manuscript and similarity index of the manuscript.

## Source of funding

We acknowledge sponsorship of this study by a 2024 Dana Masyarakat Research Grant from the Faculty of Dentistry, Universitas Gadjah Mada Yogyakarta, Indonesia, under Contract No. 3890/UN1/KG/Set.KG1/LT/2024.

## Conflict of interest

The authors have no conflict of interest to declare.
